# Predictive value of insulin resistance surrogates for the development of diabetes in individuals with baseline normoglycemia: findings from two independent cohort studies in China and Japan

**DOI:** 10.1186/s13098-024-01307-x

**Published:** 2024-03-16

**Authors:** Qing Shangguan, Qiuling Liu, Ruijuan Yang, Shuhua Zhang, Guotai Sheng, Maobin Kuang, Yang Zou

**Affiliations:** 1 Jiangxi Provincial Geriatric Hospital, Jiangxi Provincial People’s Hospital, The First Affiliated Hospital of Nanchang Medical College, Nanchang, 330006 Jiangxi China; 2 Cardiovascular Research Institute of Jiangxi Province, Jiangxi Provincial People’s Hospital, The First Affiliated Hospital of Nanchang Medical College, Nanchang, 330006 Jiangxi China; 3 Jiangxi Medical College, Nanchang University, Nanchang, 330006 Jiangxi China; 4 Department of Endocrinology, Jiangxi Provincial People’s Hospital, The First Affiliated Hospital of Nanchang Medical College, Nanchang, 330006 Jiangxi China

**Keywords:** Insulin resistance surrogates, Diabetes, IR surrogates, Triglycerides glucose index, TyG-BMI, TG/HDL-C ratio, MetS-IR

## Abstract

**Background:**

Insulin resistance (IR) plays a crucial role in the occurrence and progression of diabetes. This study aimed to evaluate and compare the predictive value of four IR surrogates, including the triglycerides glucose (TyG) index, TyG and body mass index (TyG-BMI), triglycerides/high-density lipoprotein cholesterol (TG/HDL-C) ratio, and the metabolic score for IR (MetS-IR) for diabetes in two large cohorts.

**Methods:**

A total of 116,661 adult participants from the China Rich Healthcare Group and 15,464 adult participants from the Japanese NAGALA cohort were included in the study. Multivariable Cox proportional hazards models were used to assess the standardized hazard ratio (HR) of the TyG index, TyG-BMI, TG/HDL-C ratio, and MetS-IR directly associated with diabetes. Receiver operating characteristic (ROC) curve and time-dependent ROC curve analysis were performed to evaluate and compare the predictive value of the four IR surrogates for diabetes.

**Results:**

In the two independent cohorts, the average follow-up time was 3.1 years in the China cohort, with 2681(2.30%) incident cases of diabetes recorded, and 6.13 years in the Japan cohort, with 373 incident cases (2.41%) of diabetes recorded. After adjusting for potential confounding factors, we found that among the four IR surrogates, TyG-BMI and MetS-IR showed stronger associations with diabetes. The stronger associations persisted even after further stratification by age, sex, hypertension, and obese subgroups. In terms of diabetes prediction, based on ROC analysis, TyG-BMI demonstrated the highest predictive accuracy for diabetes in the Chinese population, while both TyG-BMI and MetS-IR showed the highest predictive accuracy in the Japanese population. The results of further subgroup ROC analysis confirmed the robustness of these findings. Furthermore, the time-dependent ROC results indicated that among the four IR surrogates, MetS-IR exhibited the highest accuracy in predicting future diabetes at various time intervals in the Japanese population.

**Conclusion:**

Our findings suggest that evaluating TyG-BMI and MetS-IR as IR surrogates may be the most useful for predicting diabetes events and assessing the risk of developing diabetes in East Asian populations.

**Supplementary Information:**

The online version contains supplementary material available at 10.1186/s13098-024-01307-x.

## Background

Diabetes is one of the most prominent health issues globally, with high incidence and prevalence rates. It is a key risk factor contributing to increased cardiovascular disease, physical disability, and mortality rates [1–3]. According to the latest survey report released by the International Diabetes Federation, as of 2021, there were 536.6 million adults (aged 21–79) worldwide with diabetes, and the number is projected to further increase to 783 million by 2045, representing a 45.92% increase [4]. In addition, diabetes has a significant and growing economic impact. The reported cost of diabetes-related diseases was approximately $966 billion in 2021, and it is estimated to reach $1054 billion by 2045, exerting tremendous pressure on families and healthcare systems [4]. Given the high prevalence of diabetes and its increasing burden, early identification of effective screening strategies to identify high-risk populations is crucial for reducing diabetes incidence rates.

IR is a critical characteristic preceding the development of diabetes [5, 6], and early quantification of IR is essential for reducing the incidence of diabetes. Various methods have been established for assessing IR, including the gold standard hyperinsulinemic-euglycemic clamp test [7], non-invasive homeostasis model assessment of IR (HOMA-IR), and quantitative insulin sensitivity check index (QUICKI) [8, 9], as well as several IR surrogates that involve combining simple parameters such as the TyG index, TyG-BMI, TG/HDL-C ratio, and MetS-IR [10–13]. Taken together, each of these IR assessment methods has its own advantages. However, from an epidemiological perspective, the invasive nature and complexity of the hyperinsulinemic-euglycemic clamp test may limit its widespread use in population-based surveys [14]. The non-invasive nature of HOMA-IR and QUICKI offers certain advantages, but it is important to note that the measurement of serum insulin is not routine [15, 16], and the insulin-based assessment methods yield fewer ideal results in populations with impaired β-cell function [8]. On the other hand, most IR surrogates are combinations of simple parameters, which do not require insulin quantification and offer convenience in measurement. These notable advantages make IR surrogates more suitable for epidemiological research and clinical practice in diabetes. Recent studies have provided compelling statistical evidence demonstrating the significant advantages of insulin resistance (IR) substitutes in assessing the risk and predicting the onset of diabetes. Furthermore, an increasing number of studies are employing individual lipid and blood glucose level indicators, along with their combined values, to develop predictive models for metabolic syndrome and diabetes, as well as other IR-related diseases [13, 17–20]. However, there is limited research systematically comparing the differences among IR surrogates in diabetes prediction and risk assessment capabilities, and the author believed that identifying one or two of the most recommended IR surrogates for diabetes prediction may be very useful for clinical practice or epidemiological investigations. Therefore, the current study aimed to evaluate and compare the predictive value of four IR surrogates, namely the TyG index, TyG-BMI, TG/HDL-C ratio, and MetS-IR, for diabetes based on national health examination data from the Rich Healthcare Group and the NAGALA cohort data from Japan.

## Methods

### Study data and population

**Fig. 1:**
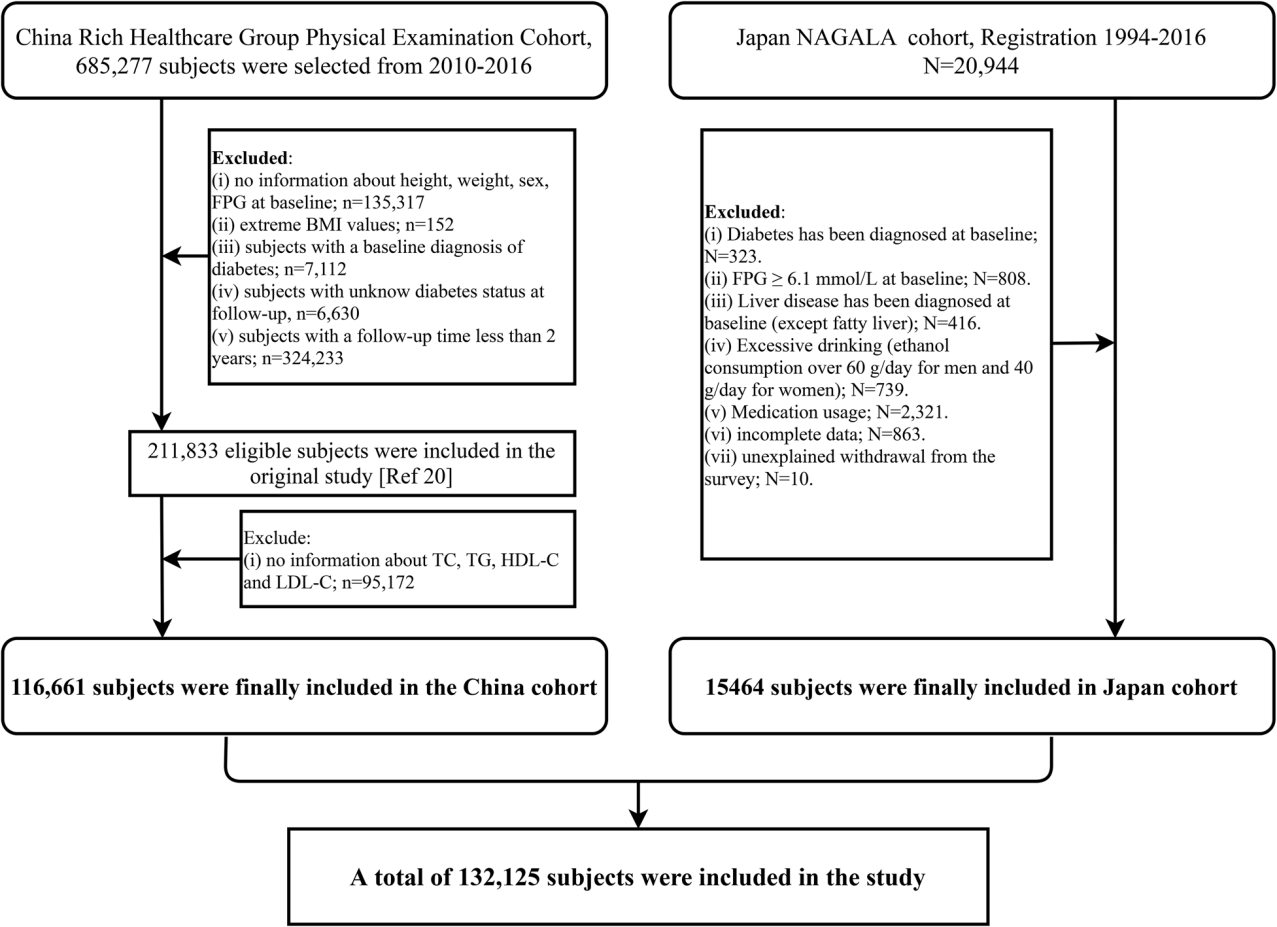
Flow chart for inclusion and exclusion of study participants

This study utilized data from two population-based cohorts: the Rich Healthcare Group cohort in China and the NAGALA cohort in Japan. Detailed information regarding the design and methods of these cohorts has been previously published [21, 22], and the research data has been deposited in the public database DRYAD for open access [23, 24]. In brief, both the Rich Healthcare Group cohort and the NAGALA cohort are longitudinal follow-up studies conducted among individuals undergoing routine health check-ups. The Rich Healthcare Group cohort recruited participants who underwent health examinations in 11 cities in China between 2011 and 2016, while the NAGALA cohort recruited participants who underwent health examinations at the Murakami Memorial Hospital in Japan between 1994 and 2016. Building upon previous research, the current study aimed to further investigate the predictive value of four IR surrogates, namely the TyG index, TyG-BMI, TG/HDL-C ratio, and MetS-IR, for diabetes. Figure 1 illustrates the participant selection process for this study.

### Ethics approval and informed consent

The research protocol for the Rich Healthcare Group cohort has been approved by their institutional review board [21]. The NAGALA cohort also has obtained approval from the Murakami Memorial Hospital ethics committee [22]. The current study was a post-hoc analysis of data from these two cohort studies, and the research protocol was approved by the Institutional Ethics Committee of the authors' institution (Ethics Committee of Jiangxi Provincial People’s Hospital). Additionally, since the research data were anonymized, the informed consent requirement for participants was waived by the Jiangxi Provincial People’s Hospital Ethics Committee. The entire study process was conducted in accordance with the principles of the Helsinki Declaration and followed the guidelines for Strengthening the Reporting of Observational Studies in Epidemiology (STROBE) statement (Additional file 3: Text S1).

### Collection and measurements of baseline information

In both cohorts, the baseline information of the participants was recorded using standardized questionnaires administered by trained healthcare professionals. This information included demographic information (age, gender), clinical characteristics (height, weight, blood pressure), living habits (smoking status, drinking status), and other relevant factors. Blood pressure, height, and weight were measured indoors using standard procedures. Fasting venous blood samples were collected from the participants after at least 8 h of overnight fasting and analyzed using automated biochemical analyzers in standard laboratories to measure biochemical parameters, including alanine aminotransferase (ALT), aspartate aminotransferase (AST), fasting plasma glucose (FPG), HDL-C, low-density lipoprotein cholesterol (LDL-C), TG, and total cholesterol (TC). Note: The LDL-C concentration in the NAGALA cohort was calculated using the Friedewald equation [25].

### Calculation of IR surrogates

The four IR surrogates were calculated as follows:

TyG index = ln [TG (mg/dL) × FPG (mg/dL)/2] [10]

TyG-BMI = TyG index × BMI [11]

TG/HDL-C ratio = TG (mmol/L)/HDL-C (mmol/L) [12]

MetS-IR = Ln [(2 × FPG (mg/dL)) + TG (mg/dL)] × BMI (kg/m ^2^) / (Ln [HDL-C (mg/dL)]) [13]

### Predefined subgroups

To explore whether the predictive value of the four IR surrogates for diabetes varied across different population subgroups, several predefined subgroups were established based on sex, age, hypertension status, and BMI, representing commonly observed phenotypes.

(i) Sex subgroups were divided into males and females based on physiological characteristics.

(ii) The obese subgroup in both the Chinese cohort and Japanese cohort was determined based on the clinical cutoff values for BMI established by the Chinese Obesity Working Group and the Japanese Diabetes Society. Participants were categorized into the obese group (Chinese cohort: BMI ≥ 28 kg/m^2^; Japanese cohort: BMI ≥ 25 kg/m^2^) and the non-obese group (Chinese cohort: BMI < 28 kg/m^2^; Japanese cohort: BMI < 25 kg/m^2^) [26–28].

(iii) Age subgroups: Considering that the average age of natural menopause in Asian women is around 50 years [29], and 50 years is a critical age for the development of metabolic-related diseases in both sexes [30], the cutoff point for the age subgroup in the current study was set at 50 years.

(iv) Hypertension subgroups were defined according to individuals with or without baseline hypertension. Hypertension was diagnosed as baseline measurements of systolic blood pressure (SBP) ≥ 140 mmHg or diastolic blood pressure (DBP) ≥ 90 mmHg [31].

### Definition of diabetes

In both cohorts, the diabetes status was determined based on repeated health examinations conducted annually. The diagnosis of diabetes followed the criteria outlined in the American Diabetes Association guidelines [32], which included blood glucose measurements (FPG ≥ 7.0 mmol/L or HbA1c ≥ 6.5%) during follow-up visits and other physician-diagnosed cases of diabetes.

### Statistical analysis

Cox proportional hazards regression models were established to derive the HRs and 95% confidence intervals (CIs) for the associations between the four IR surrogates and diabetes. To eliminate the dimensionality and magnitude differences of the four IR surrogates in calculating HR, we standardized these surrogates using Z-scores before incorporating them into the Cox regression models. The non-normally distributed TG/HDL-C ratio was logarithmically transformed to achieve a normal distribution before Z-score standardization. Prior to the formal regression analysis, we assessed the variance inflation factor through multiple linear regression to evaluate whether collinearity existed between the four IR surrogates and other baseline variables [33]. Additionally, we used Kaplan–Meier plots and log-rank tests to verify whether the inclusion of IR surrogates alone in the model complied with the proportional hazards assumption [34].

In the regression analysis, we first used an unadjusted univariate Cox proportional hazards regression model to initially explore the association between all baseline indicators and diabetes risk; subsequently, based on the results of univariate Cox regression analysis and following the recommendations of the STROBE guidelines, three sequentially adjusted multivariable Cox regression models were established to assess the associations between four IR surrogates and diabetes risk in the entire study population, as well as separately in the Chinese or Japanese populations [35]. Model 1 adjusted for potential confounders, including sex, age, race, and height. In the subsequent adjustment strategy (Model 2), we considered the influence of SBP, DBP, and smoking status. Finally, in Model 3, we further adjusted for blood glucose parameters (FPG), lipid parameters (HDL-C and LDL-C), and liver enzyme parameters (ALT) based on Model 2. Additionally, we calculated the corresponding E-values based on the HR and 95%CI of each IR surrogate in the Model 3 to explore the potential impact of unmeasured confounders on the association between these IR surrogates and diabetes risk. The E-value quantifies the degree of association that unmeasured confounders would need to have with the risk of diabetes to negate the observed associations [36]. After establishing the significant associations between the four IR surrogates and diabetes, we proceeded to calculate and compare the area under the curve (AUC) for these four IR surrogates in predicting diabetes using ROC analysis and the DeLong test in both the Chinese and Japanese populations [37]. Furthermore, we conducted time-dependent ROC analysis in the Japanese population to explore the predictive performance of TyG index, TyG-BMI, TG/HDL-C ratio, and MetS-IR for future diabetes at various time intervals.

To explore whether the correlation/predictive value of the four IR surrogates with diabetes varied across different populations, we performed the same analysis steps in predefined subgroups using a multivariable Cox regression model (Model 3) and ROC analysis. All statistical analyses were conducted using R software version 3.4.3 and Empower Stats 2.0 software. Two-sided tests were used, and statistical significance was defined as *P* < 0.05.

## Results

### Baseline characteristics analysis of two cohorts

A total of 132,125 participants were included in this study, with 88.3% of the study population from the Chinese cohort and the remaining 11.7% from the Japanese cohort. The baseline characteristics of the two cohort populations were summarized in Table 1. Overall, the distribution and proportions of most baseline characteristics were comparable between the Chinese and Japanese cohorts, such as age (median age 41 vs 42), sex composition (male 53.80% vs 54.51%; female 46.20% vs 45.49%), height, ALT and HDL-C levels, and the proportion of drinkers. However, there were some differences between the two cohorts in certain measured parameters and biochemical indicators. It can be observed that, compared to the Japanese population, the Chinese population generally had higher weight and BMI, higher baseline levels of SBP, DBP, TG, AST, and the four IR surrogates, and relatively lower levels of FPG, TC, and LDL-C.

**Table 1:** Characteristics regarding the study variables at baseline in two cohort studies

	Chinese cohort	Japanese cohort
No. of subjects	116,661	15,464
Age (years)	41.00 (34.00–53.00)	42.00 (37.00–50.00)
Sex		
Male	62,759 (53.80%)	8430 (54.51%)
Female	53,902 (46.20%)	7034 (45.49%)
Height (cm)	166.29 (8.31)	165.12 (8.47)
Weight (kg)	64.88 (12.11)	60.64 (11.62)
BMI (kg/m^2^)	23.35 (3.30)	22.12 (3.13)
SBP (mmHg)	119.43 (16.68)	114.50 (14.97)
DBP (mmHg)	74.44 (10.98)	71.58 (10.50)
FPG (mmol/L)	4.95 (0.61)	5.16 (0.41)
TC (mmol/L)	4.79 (0.90)	5.13 (0.86)
TG (mmol/L)	1.10 (0.76–1.66)	0.73 (0.50–1.12)
HDL-C (mmol/L)	1.35 (1.16–1.56)	1.41 (1.16–1.71)
LDL-C (mmol/L)	2.70 (2.29–3.16)	3.04 (2.56–3.57)
ALT (IU/L)	18.00 (13.00–27.50)	17.00 (13.00–23.00)
AST (IU/L)	22.00 (18.60–26.90)	17.00 (14.00–21.00)
TyG index	8.41 (0.61)	8.03 (0.65)
TyG-BMI	197.29 (36.96)	178.60 (34.54)
TG/HDL-C ratio	0.82 (0.52–1.34)	0.51 (0.30–0.91)
MetS-IR	33.82 (6.61)	31.16 (6.50)
Drinking status		
No	26,237 (22.49%)	11,805 (76.34%)
Yes	6396 (5.48%)	3659 (23.66%)
Not recorded	84,028 (72.03%)	0 (0.00%)
Smoking status		
No	24,649 (21.13%)	9031 (58.40%)
Yes	7984 (6.84%)	6433 (41.60%)
Not recorded	84,028 (72.03%)	0 (0.00%)

The data of continuous variables were described by means and standard deviations. Categorical variables were expressed as proportions

*BMI* Body mass index, *SBP* systolic blood pressure, *DBP* diastolic blood pressure, *FPG* fasting plasma glucose, *TC* total cholesterol, *HDL-C* high-density lipoprotein cholesterol, *LDL-C* low-density lipid cholesterol, *TG* triglyceride, *TyG index* the triglyceride-glucose index, *TyG-BMI* triglyceride glucose-body mass index, *TG/HDL-C ratio* triglyceride/high-density lipoprotein cholesterol ratio, *METS-IR* metabolic score for insulin resistance, *ALT* alanine aminotransferase, *AST* aspartate aminotransferase

### Standardized HRs for the associations between the four IR surrogates and diabetes

In the two independent cohorts, the average follow-up time was 3.1 years (maximum 7.56 years) in the Chinese cohort, with 2681 incident cases (2.30%) of diabetes recorded, and 6.13 years (maximum 13.14 years) in the Japanese cohort, with 373 incident cases (2.41%) of diabetes recorded.

In the screening for collinearity between the four IR surrogates and baseline variables (Additional file 1: Tables S1–4), we identified that weight, BMI, TC, TG, and drinking status were collinear and were not included in the subsequent multivariable models. Furthermore, the Kaplan–Meier curve analysis, incorporating follow-up time and exposure variables, indicated that the Cox proportional hazards models constructed to assess the associations between the four IR surrogates and diabetes were appropriate for this study (Additional file 1: Figures S1–4).

The results of univariate Cox regression analysis indicated that, except for drinking status, all other baseline indicators were significantly associated with the risk of diabetes. Among them, having a smoking habit and higher levels of age, height, weight, BMI, SBP, DBP, FPG, TC, TG, LDL-C, ALT, AST were significantly associated with an increased risk of diabetes. Conversely, higher levels of HDL-C and being female were associated with a lower risk of diabetes (Additional file 1: Table S5). Subsequently, the independent associations between the IR surrogates and diabetes were demonstrated in three sequentially adjusted multivariable Cox regression models (Table 2). Consistent with the majority of previous findings, after adjusting for potential confounders, all four IR surrogates showed a significant positive association with diabetes. Based on the standardized HRs, TyG-BMI and MetS-IR exhibited the strongest associations with diabetes among the four IR surrogates (Model 3, HR: TyG-index 1.33 vs TyG-BMI 1.51 vs TG/HDL-C ratio 1.37 vs MetS-IR 1.51). Furthermore, after stratifying by ethnicity, this stronger association was still observed in the Chinese population (Model 3, HR: TyG-index 1.33 vs TyG-BMI 1.48 vs TG/HDL-C ratio 1.37 vs MetS-IR 1.47) and the Japanese population (Model 3, HR: TyG-index 1.23 vs TyG-BMI 1.79 vs TG/HDL-C ratio 1.26 vs MetS-IR 1.86). These findings suggested that both TyG-BMI and MetS-IR may be more useful in assessing the risk of diabetes, whether in the Chinese or Japanese population.

**Table 2:** Cox regression analyses for the association between TyG index, TyG-BMI, TG/HDL-C ratio, MetS-IR and incident DM in different models

	Hazard ratios (95% confidence interval) (Per SD increase)			
	TyG index	TyG-BMI	TG/HDL-C ratio	MetS-IR
All cohort				
Model 1	2.09 (2.02, 2.16)	**2.17 (2.10, 2.24)**	1.65 (1.59, 1.71)	**1.92 (1.86, 1.98)**
Model 2	2.00 (1.93, 2.07)	**2.09 (2.02, 2.16)**	1.57 (1.51, 1.63)	**1.84 (1.78, 1.90)**
Model 3	1.33 (1.28, 1.38)	**1.51 (1.45, 1.57)**	1.37 (1.31, 1.44)	**1.51 (1.45, 1.57)**
	E value = 1.99	E value = 2.39	E value = 2.08	E value = 2.39
Races (model 1)				
Chinese cohort	2.09 (2.02, 2.17)	**2.13 (2.06, 2.20)**	1.61 (1.55, 1.67)	**1.86 (1.80, 1.92)**
Japanese cohort	2.15 (1.93, 2.40)	**2.54 (2.31, 2.79)**	1.94 (1.76, 2.15)	**2.38 (2.19, 2.60)**
Races (model 2)				
Chinese cohort	2.01 (1.93, 2.08)	**2.05 (1.98, 2.12)**	1.54 (1.48, 1.60)	**1.78 (1.73, 1.85)**
Japanese cohort	2.03 (1.81, 2.26)	**2.54 (2.28, 2.83)**	1.85 (1.67, 2.04)	**2.33 (2.12, 2.57)**
Races (model 3)				
Chinese cohort	1.33 (1.28, 1.39)	**1.48 (1.42, 1.54)**	1.37 (1.31, 1.44)	**1.47 (1.41, 1.53)**
	E value = 1.99	E value = 2.32	E value = 2.08	E value = 2.3
Japanese cohort	1.23 (1.07, 1.40)	**1.79 (1.57, 2.04)**	1.26 (1.08, 1.47)	**1.86 (1.62, 2.13)**
	E value = 1.76	E value = 2.98	E value = 1.83	E value = 3.12

Model 1 adjusted for gender, age, race and height

Model 2 adjusted for gender, age, race, height, smoking status, SBP and DBP

Model 3 adjusted for gender, age, race, height, smoking status, SBP, DBP, FPG, HDL-C, LDL-C and ALT

Race variables are not adjusted in the grouping of races

Furthermore, to validate the stability of the aforementioned association analysis results, we calculated the E-values for each IR surrogate based on Model 3. In the All cohort, the E-values for TyG index, TyG-BMI, TG/HDL-C ratio, and MetS-IR were 1.99, 2.39, 2.08, and 2.39, respectively. Upon further differentiation by ethnicity, in the Chinese population, the E-values for TyG index, TyG-BMI, TG/HDL-C ratio, and MetS-IR were 1.99, 2.32, 2.08, and 2.3, respectively; in the Japanese population, the E-values for TyG index, TyG-BMI, TG/HDL-C ratio, and MetS-IR were 1.76, 2.98, 1.83, and 3.12, respectively. We observed that the E-values for these IR surrogates were all relatively large and higher than the corresponding HR values, indicating that it is unlikely that unmeasured confounders were affecting the stability of the results.

### Association analysis of the four IR surrogates with diabetes in predefined subgroups

In predefined subgroups representing common phenotypes, we further explored the associations between the four IR surrogates and diabetes in the Chinese and Japanese populations. Detailed results were shown in Table 3. Across all subgroups, a consistent finding was observed: TyG-BMI and MetS-IR had the highest associations with diabetes among the four IR surrogates, which aligned with the results obtained from the overall population analysis and the analysis stratified by ethnicity (Table 2). This finding further supported the notion that TyG-BMI and MetS-IR may be the strongest IR surrogates for assessing the risk of diabetes.

**Table 3:** Stratified association between TyG index, TyG-BMI, TG/HDL-C ratio, MetS-IR and diabetes by age, BMI, sex, and hypertension

	Hazard ratios (95% confidence interval) (Per SD increase)	
	Chinese cohort	Japanese cohort
Sex (male)		
TyG index	1.28 (1.22, 1.34)	1.14 (0.98, 1.32)
TyG-BMI	**1.45 (1.38, 1.52)**	**1.62 (1.38, 1.90)**
TG/HDL-C ratio	1.31 (1.24, 1.38)	1.17 (0.99, 1.38)
MetS-IR	**1.44 (1.38, 1.52)**	**1.71 (1.45, 2.03)**
Sex (female)		
TyG index	1.40 (1.28, 1.52)	1.51 (1.10, 2.07)
TyG-BMI	**1.47 (1.36, 1.58)**	**2.12 (1.63, 2.74)**
TG/HDL-C ratio	1.10 (1.04, 1.16)	1.35 (1.01, 1.80)
MetS-IR	**1.43 (1.32, 1.55)**	**2.19 (1.65, 2.89)**
Non-obese		
TyG index	1.33 (1.27, 1.40)	1.18 (0.99, 1.42)
TyG-BMI	**1.61 (1.50, 1.72)**	**1.30 (0.98, 1.72)**
TG/HDL-C ratio	1.37 (1.30, 1.45)	1.21 (0.99, 1.48)
MetS-IR	**1.59 (1.49, 1.71)**	**1.31 (0.96, 1.81)**
Obese		
TyG index	1.15 (1.05, 1.26)	1.16 (0.94, 1.42)
TyG-BMI	**1.54 (1.45, 1.62)**	**1.90 (1.52, 2.39)**
TG/HDL-C ratio	1.35 (1.29, 1.41)	1.18 (0.94, 1.49)
MetS-IR	**1.50 (1.42, 1.58)**	**1.97 (1.57, 2.49)**
Age (≥ 50 years)		
TyG index	1.22 (1.16, 1.28)	1.14 (0.92, 1.42)
TyG-BMI	**1.30 (1.23, 1.37)**	**1.49 (1.15, 1.92)**
TG/HDL-C ratio	1.24 (1.17, 1.31)	1.16 (0.91, 1.48)
MetS-IR	**1.28 (1.22, 1.36)**	**1.51 (1.15, 1.98)**
Age (< 50 years)		
TyG index	1.44 (1.33, 1.55)	1.25 (1.05, 1.48)
TyG-BMI	**1.74 (1.62, 1.86)**	**1.79 (1.53, 2.10)**
TG/HDL-C ratio	1.50 (1.38, 1.64)	1.29 (1.06, 1.56)
MetS-IR	**1.76 (1.63, 1.89)**	**1.86 (1.58, 2.19)**
Hypertension (no)		
TyG index	1.36 (1.29, 1.43)	1.28 (1.11, 1.49)
TyG-BMI	**1.51 (1.44, 1.59)**	**1.87 (1.63, 2.15)**
TG/HDL-C ratio	1.39 (1.32, 1.47)	1.33 (1.13, 1.56)
MetS-IR	**1.49 (1.42, 1.57)**	**1.99 (1.71, 2.30)**
Hypertension (yes)		
TyG index	1.26 (1.18, 1.35)	1.03 (0.73, 1.44)
TyG-BMI	**1.38 (1.29, 1.47)**	**1.44 (1.05, 1.97)**
TG/HDL-C ratio	1.29 (1.20, 1.40)	1.03 (0.70, 1.50)
MetS-IR	**1.38 (1.29, 1.49)**	**1.50 (1.08, 2.08)**

Abbreviations as in Table 1. Models adjusted for the same covariates as in model 3 (Table 2), except for the stratification variable

### ROC analysis to evaluate the predictive value of the four IR surrogates for diabetes

**Fig. 2:**
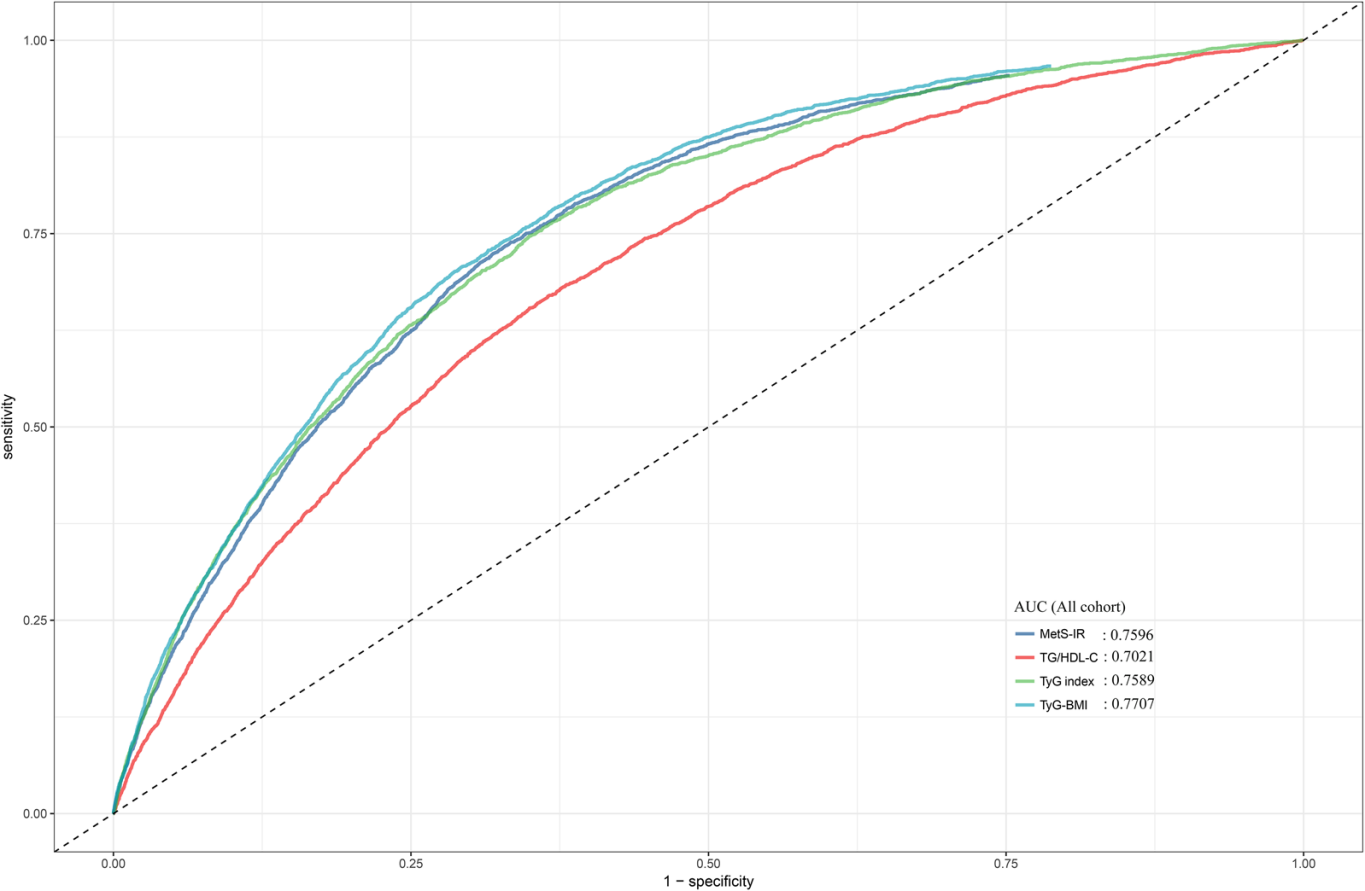
ROC curve for predicting diabetes using TyG index, TyG-BMI, TG/HDL-C ratio, MetS-IR. *TyG index* the triglyceride-glucose index, *TyG-BMI* triglyceride glucose-body mass index, *TG/HDL-C ratio* triglyceride/high-density lipoprotein cholesterol ratio, *METS-IR* metabolic score for insulin resistance

**Fig. 3:**
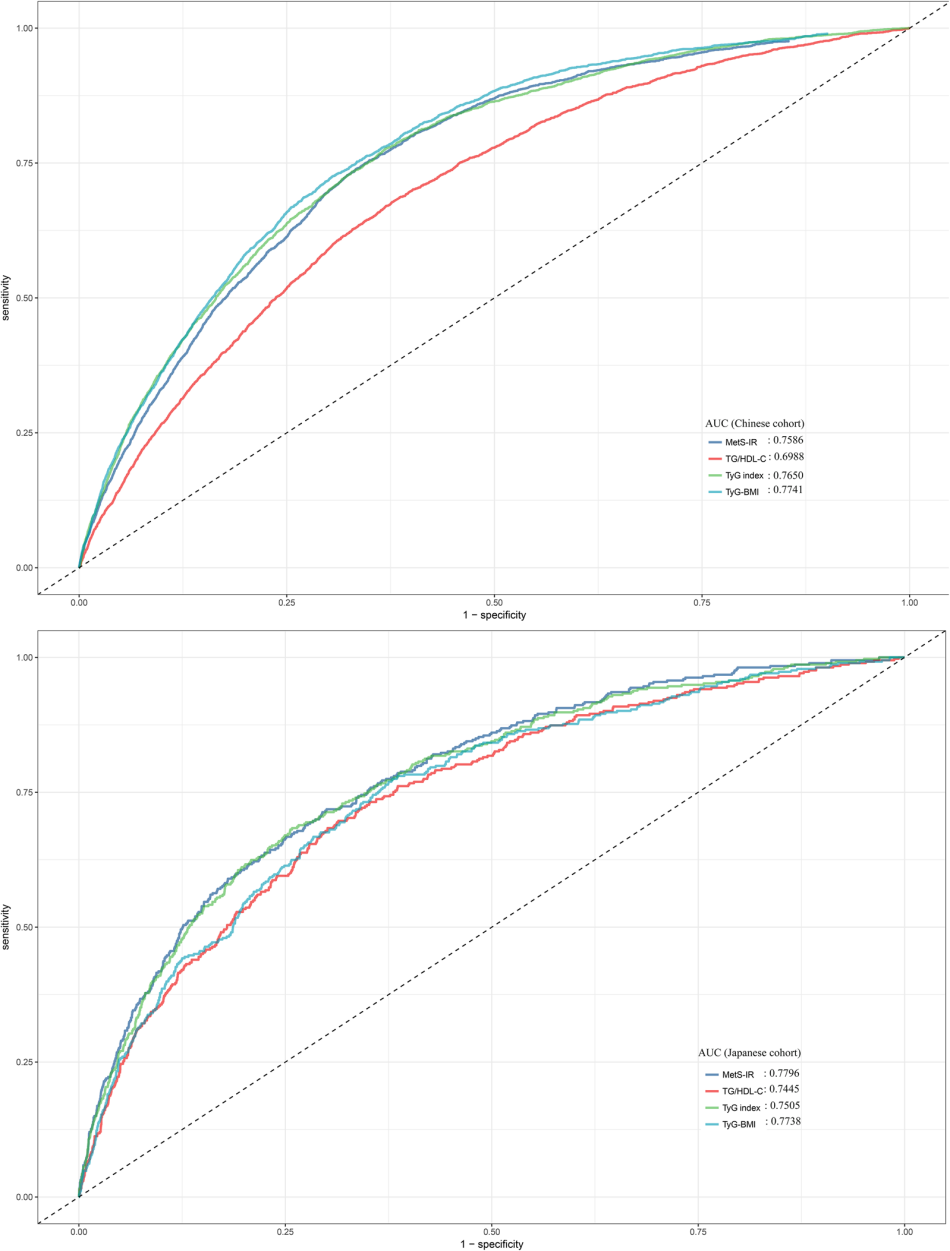
ROC curves for predicting diabetes using TyG index, TyG-BMI, TG/HDL-C ratio, MetS-IR in Chinese cohort and Japanese cohort. *TyG index* the triglyceride-glucose index, *TyG-BMI* triglyceride glucose-body mass index, *TG/HDL-C ratio* triglyceride/high-density lipoprotein cholesterol ratio, *METS-IR* metabolic score for insulin resistance

Table 4 presents the AUC values of the four IR surrogates for predicting diabetes. It can be observed that in the overall population (Fig. 2), TyG-BMI had the highest AUC value for predicting diabetes compared with other IR surrogates (0.7707, all DeLong test *P* < 0.05), followed by MetS-IR (0.7596), TyG-index (0.7589), and TG/HDL-C ratio (0.7021). After stratifying by ethnicity, we found that in the Chinese population, TyG-BMI had the highest AUC value (0.7741, all DeLong test *P* < 0.05), while in the Japanese population, although TyG-BMI and MetS-IR had relatively higher AUC values (0.7738 and 0.7796) (Fig. 3), after performing the DeLong test for comparison, we found that they were only significantly higher than TG/HDL-C ratio, with no statistically significant difference from TyG index.

**Table 4:** Predictive value of four IR surrogates for the diabetes

	AUC	95%CI low	95%CI up	Best threshold	Specificity	Sensitivity
All cohort						
TyG index	0.7589^*^	0.7507	0.7672	8.5654	0.6509	0.7466
TyG-BMI	**0.7707**	0.7628	0.7786	212.6980	0.7082	0.7063
TG/HDL-C ratio	0.7021^*^	0.6931	0.7111	1.0237	0.6503	0.6539
MetS-IR	0.7596^*^	0.7515	0.7677	35.9734	0.6811	0.7243
Race (Chinese)						
TyG index	0.7650^*^	0.7564	0.7736	8.5673	0.6312	0.7766
TyG-BMI	**0.7741**	0.7658	0.7824	213.2966	0.6954	0.7251
TG/HDL-C ratio	0.6988^*^	0.6893	0.7084	1.0090	0.6236	0.6774
MetS-IR	0.7586^*^	0.7501	0.7672	35.6670	0.6502	0.7553
Race (Japanese)						
TyG index	0.7505	0.7255	0.7754	8.1960	0.6198	0.7748
TyG-BMI	**0.7738**	0.7498	0.7979	197.2987	0.7416	0.6836
TG/HDL-C ratio	0.7445^*^	0.7192	0.7698	0.7505	0.6858	0.6971
MetS-IR	**0.7796**	0.7562	0.8029	33.6338	0.7001	0.7185

*AUC* area under the curve; other abbreviations as in Table 1

^*^: Delong test *P* < 0.05 compared with TyG-BMI

### Analysis of the predictive value of the four IR surrogates for diabetes in predefined subgroups

To evaluate whether the predictive value of the four IR surrogates for diabetes varied across different populations, we further conducted ROC analysis in predefined subgroups. Detailed results are presented in Table 5. Overall, consistent with the findings from the stratification by ethnicity in Table 4, we observed similar patterns in the ROC analysis of the subgroups. In the Chinese cohort, TyG-BMI demonstrated the best predictive value for diabetes in almost all subgroups, with statistically significant differences observed in the majority of AUC values after conducting the DeLong test for comparison; while in the Japanese cohort, MetS-IR had the highest AUC values for predicting diabetes in all subgroups, followed by TyG-BMI. However, the differences between the two were statistically insignificant in almost all subgroups, and only in the male subgroup, obese subgroup, age < 50 years subgroup, and no hypertension subgroup, both MetS-IR and TyG-BMI exhibited significantly higher AUC values than TyG index and TG/HDL-C ratio. Additionally, in both cohorts, the predictive accuracy for predicting diabetes was higher in females compared to males, in non-obese individuals compared to obese individuals, in younger individuals (Age < 50 years) compared to older individuals (Age ≥ 50 years), and in individuals with normal baseline blood pressure compared to those with hypertension.

**Table 5:** The best threshold, sensitivities, specificities, and area under the curve of four IR surrogates for predicting diabetes in different subgroups

	Chinese cohort				Japanese cohort			
	AUC	Best threshold	Specificity	Sensitivity	AUC	Best threshold	Specificity	Sensitivity
Sex (male)								
TyG index	0.7221^*^	8.7791	0.6470	0.6859	0.6966^*^	8.7670	0.8002	0.5105
TyG-BMI	**0.7356**	220.2488	0.6465	0.7145	**0.7251**	206.1369	0.7113	0.6573
TG/HDL-C ratio	0.6454^*^	1.1398	0.5567	0.6679	0.6936^*^	0.8178	0.5687	0.7448
MetS-IR	0.7186^*^	36.7735	0.5754	0.7532	0.7346^*^	37.6406	0.7627	0.6154
Sex (female)								
TyG index	0.7996	8.4446	0.7030	0.7831	0.7934	8.0568	0.7346	0.7816
TyG-BMI	**0.8000**	197.4111	0.7221	0.7604	**0.8016**	180.6269	0.7858	0.7241
TG/HDL-C ratio	0.7396^*^	0.9309	0.7469	0.6204	0.7796	0.4560	0.6764	0.7931
MetS-IR	0.7842^*^	33.8494	0.7369	0.7352	**0.8001**	29.8919	0.7256	0.7471
Non-obese								
TyG index	0.7622	8.5659	0.6598	0.7457	**0.7269**	8.2027	0.6830	0.6667
TyG-BMI	0.7653	202.0524	0.6430	0.7603	**0.7238**	179.9124	0.6782	0.6617
TG/HDL-C ratio	0.6938^*^	1.0237	0.6601	0.6341	0.7150	0.7524	0.7477	0.5871
MetS-IR	0.7457^*^	34.6257	0.6482	0.7392	**0.7314**	31.2083	0.6762	0.6667
Obese								
TyG index	0.6497	8.8756	0.5268	0.7057	0.6531^*^	8.7738	0.6926	0.5523
TyG-BMI	0.6440	272.1837	0.6726	0.5476	**0.6898**	8.7738	0.6926	0.5523
TG/HDL-C ratio	0.5712^*^	1.8093	0.6730	0.4348	0.6494^*^	1.2743	0.6543	0.5698
MetS-IR	0.6131^*^	44.9568	0.5091	0.6603	**0.7049**	41.7010	0.6330	0.6860
Age (≥ 50 years)								
TyG index	0.6780^*^	8.7798	0.6351	0.6276	0.6747	8.5186	0.7224	0.5396
TyG-BMI	**0.6927**	219.2980	0.6528	0.6406	**0.6889**	198.2597	0.7009	0.6043
TG/HDL-C ratio	0.6146^*^	0.9931	0.5048	0.6680	0.6741	1.1047	0.7813	0.4748
MetS-IR	0.6835^*^	36.1923	0.5831	0.7070	**0.7012**	35.5029	0.7451	0.5468
Age (< 50 years)								
TyG index	0.8099^*^	8.6016	0.7122	0.7835	0.7776^*^	8.1960	0.6600	0.7991
TyG-BMI	**0.8272**	214.0949	0.7489	0.7795	**0.8094**	192.9588	0.7241	0.7692
TG/HDL-C ratio	0.7607^*^	1.1092	0.7142	0.7025	0.7716^*^	0.7505	0.7132	0.7350
MetS-IR	0.8175^*^	36.0175	0.7099	0.7942	**0.8143**	33.7494	0.7235	0.7650
Hypertension (No)								
TyG index	0.7719^*^	8.5426	0.6524	0.7698	0.7544^*^	8.1960	0.6376	0.7695
TyG-BMI	**0.7811**	213.7032	0.7413	0.6888	**0.7752**	197.2987	0.7625	0.6667
TG/HDL-C ratio	0.7076^*^	1.0091	0.6544	0.6683	0.7465^*^	0.7747	0.7145	0.6791
MetS-IR	0.7673^*^	35.4151	0.6774	0.7468	**0.7795**	34.7236	0.7680	0.6542
Hypertension (Yes)								
TyG index	**0.6694**	8.7874	0.5699	0.6784	0.6476	8.7327	0.6996	0.5577
TyG-BMI	0.6595	219.6879	0.5193	0.7144	**0.6708**	230.2559	0.7401	0.5385
TG/HDL-C ratio	0.6023^*^	1.1509	0.5223	0.6257	0.6501	0.9933	0.5813	0.6731
MetS-IR	0.6474^*^	36.7532	0.4840	0.7359	**0.6900**	38.3738	0.6352	0.6538

*AUC* area under the curve; other abbreviations as in Table 1

^*^: Delong test *P* < 0.05 compared with TyG-BMI

### Time-dependent ROC analysis of IR surrogates for prediction of future diabetes

To further compare the predictive abilities of TyG index, TyG-BMI, TG/HDL-C ratio, and MetS-IR for future diabetes at different time intervals, we conducted time-dependent ROC analysis in the Japanese population and presented the results in Additional file 1: Table S6. By comparing the AUC values of the four IR surrogates for predicting diabetes occurrence over 1–13 years, we found that MetS-IR had the highest AUC values at almost all time points, and TyG index and TyG-BMI exhibited similar predictive abilities, while TG/HDL-C ratio had relatively weaker predictive ability, consistent with the previous analyses. Therefore, in the Japanese population, MetS-IR may be a better surrogate marker for diabetes prediction.

## Discussion

In this analysis of two diabetes cohort studies based in China and Japan, four IR surrogates, TyG index, TyG-BMI, TG/HDL-C ratio, and MetS-IR, were positively associated with diabetes. After standardizing for HRs, TyG-BMI and MetS-IR showed stronger associations with diabetes among the four IR surrogates. These stronger associations remained consistent when further stratifying by age, sex, hypertension, and BMI subgroups. Additionally, the ROC analysis of the four IR surrogates in the Chinese population showed that TyG-BMI had the highest accuracy for predicting diabetes, while in the Japanese population, both TyG-BMI and MetS-IR showed the highest predictive accuracy.

Previous studies have reported associations between individual IR surrogates and diabetes [13, 17–19], but few have systematically compared the value of different IR surrogates in assessing diabetes risk. To date, only a few studies from China have reported differences in the assessment of diabetes risk using IR surrogates, with some analyzing them as original continuous variables and others as categorical variables [19, 38–40]. Notably, two published comparative studies by Li et al. and Dong et al. utilized the China Health and Retirement Longitudinal Study for analysis. Li et al. investigated the association between quartiles of TyG index, TyG-BMI, TG/HDL-C ratio, and diabetes [19], while Dong et al. evaluated the continuous variables of TyG index, TG/HDL-C ratio, and MetS-IR in relation to diabetes [38]. Both studies found positive associations between IR surrogates and diabetes. However, due to differences in data processing, comparing the HRs of different IR surrogates and their associations with diabetes was challenging, as the scales and magnitudes of the surrogates varied. Similar data processing approaches were employed in two other published studies in Ningxia, China, and Wuyuan, China, where the associations between the TyG index, TG/HDL-C ratio, MetS-IR, and diabetes were assessed [39, 40]. In the Ningxia study, IR surrogates were analyzed based on tertiles [39], while in the Wuyuan study, they were analyzed as original continuous variables [40]. To address the issue of comparing HRs related to diabetes for IR surrogates, the current study standardized the four IR surrogates using Z-transformation before including them in the multivariable models. The analysis of the two large independent population cohorts in this study demonstrated that the TyG index, TyG-BMI, TG/HDL-C ratio, and MetS-IR were all positively associated with diabetes in both the Chinese and Japanese populations. Among them, TyG-BMI and MetS-IR showed stronger associations with diabetes, and further subgroup analysis confirmed TyG-BMI and MetS-IR as the best IR surrogates for assessing the risk of diabetes. Considering that this study standardized and compared the four IR surrogates in two large independent cohorts and consistently found TyG-BMI and MetS-IR to be the best markers for assessing diabetes risk, the results can be considered relatively reliable.

The question of which IR surrogate is most valuable for predicting diabetes has been explored in recent studies based on Chinese populations, but there is currently no consensus. Some studies supported TyG-BMI as the superior IR surrogate [19, 41], while others supported the TyG index [38, 40, 42]; for example, the study by Mo Z and colleagues found a strong correlation between the TyG index and gestational diabetes (OR = 12.923), and it demonstrated high predictive performance for future occurrences of gestational diabetes (AUC = 0.807) [42]. It should be noted that the published studies included no more than three IR surrogates, and none of them simultaneously compared the predictive value of the TyG index, TyG-BMI, TG/HDL-C ratio, and MetS-IR for diabetes. In the present study, we simultaneously assessed the predictive value of the TyG index, TyG-BMI, TG/HDL-C ratio, and MetS-IR for diabetes in two independent cohorts. The new analysis results indicated that TyG-BMI was the most accurate IR surrogate marker for predicting diabetes in the Chinese population, while in the Japanese population, both TyG-BMI and MetS-IR exhibited the highest predictive accuracy. These findings were consistent with some observations in other metabolic disease studies, including hypertension, hypertension combined with hyperuricemia, and coronary artery disease [43–45]. These findings collectively suggested that TyG-BMI and MetS-IR may serve as better predictors of metabolic-related diseases and should be considered in further research.

The subgroup analyses based on age, sex, hypertension, and BMI were noteworthy and worth emphasizing, as the results of these analyses were highly consistent with the main analysis, despite accounting for racial differences. These subgroup findings further supported the stability and reliability of the main results of this study, indicating that TyG-BMI and MetS-IR were likely to be the most useful IR surrogates in assessing diabetes risk; while TyG-BMI was recommended for the prediction of diabetic events in the Chinese population, MetS-IR and TyG-BMI were recommended for the Japanese population. Furthermore, based on subgroup ROC analysis we identified specific populations that were more suitable for the prediction of diabetic events using IR surrogates, including females, non-obese individuals, those under 50 years of age, and those with normal baseline blood pressure. The reasons behind these specific phenomena currently lack an answer, but they can be partially explained from a risk factor perspective. Generally, male gender, obesity, hypertension, and advanced age are important risk factors for diabetes [30, 46–49]. These confounding factors may additionally affect the accuracy of IR surrogates in predicting diabetes. In relatively lower-risk populations such as females, non-obese individuals, younger age groups, and those with normal blood pressure, the potential confounding effects are reduced, making IR surrogates a more significant risk factor for diabetes. Consequently, reaching the respective threshold values of IR surrogates may have higher predictive significance for diabetes in these populations.

## Strengths and limitations

Current research has several advantages: (i) This study is the first to simultaneously evaluate and compare the predictive value and risk assessment ability of several IR surrogates for diabetes, including the TyG index, TyG-BMI, TG/HDL-C ratio, and MetS-IR. (ii) IR surrogates are combinations of commonly used clinical markers that are easily accessible and computationally straightforward. The evidence from this study provided valuable reference material for diabetes prevention and clinical practice. (iii) This study is based on two large independent cohorts, and the results obtained from both cohorts were highly consistent. The high stability of these results was further confirmed through subgroup analysis, which added to the reliability of the study's conclusions.

There are some limitations that need to be mentioned: (i) The study population is derived from China and Japan, and the results may be more applicable to East Asian populations. Further research is needed to assess the generalizability of these findings to other regions. (ii) The diabetes cohorts in China and Japan did not include measurements of postprandial glucose levels, which may result in an underestimation of the true diabetes incidence. However, despite the lower disease prevalence, the study consistently obtained highly consistent findings in different populations and subgroups, which may indicate the robustness of the current results. Moreover, measuring postprandial glucose levels may not be necessary for routine medical examinations in large epidemiological surveys. (iii) The study did not differentiate between types of diabetes. Further investigation of the applicability of IR surrogates for type 1 and type 2 diabetes could enhance the quality of the current research. However, based on the integration of published data [50, 51], the evidence from the current study was likely more suitable for the prediction and risk assessment of type 2 diabetes. (iv) The current study, being a post hoc analysis based on publicly available data, faced limitations in the study database that prevented the further assessment of factors such as the use of glucocorticoids, estrogen, thiazide-type drugs, and beta-blockers among the participants, as well as their dietary habits and physical activity. Consequently, there may be some residual confounding [52]. As an alternative approach, we calculated the E-values for the IR surrogates to assess the potential impact of confounding factors on the findings of the current study. More rigorous study designs are needed in the future to further validate our findings.

## Conclusion

In this analysis of two large cohort studies in China and Japan, strong associations between TyG index, TyG-BMI, TG/HDL-C ratio, and MetS-IR with diabetes were observed, with TyG-BMI and MetS-IR showing the highest correlations with diabetes. These findings were further validated through subgroup analysis. Additionally, in terms of diabetes prediction, TyG-BMI exhibited the highest predictive value in the Chinese population, while both TyG-BMI and MetS-IR showed the highest predictive value in the Japanese population.

### Supplementary Information

**Additional file 1: Table S1.** Collinearity diagnostics steps of TyG index with other covariates. **Table S2.** Collinearity diagnostics steps of TyG-BMI with other covariates. **Table S3.** Collinearity diagnostics steps of TG/HDL-C ratio with other covariates. **Table S4.** Collinearity diagnostics steps of MetS-IR with other covariates. **Table S5.** Univariate Cox regression analysis between all baseline indicators and diabetes risk. **Table S6.** Best threshold and areas under the time-dependent receiver operating characteristic curves for TyG index, TyG-BMI, TyG-WC, MetS-IR, TG/HDL-C ratio predicting future diabetes risk in the Japanese cohort.

**Additional file 2: Figure S1.** The cumulative hazard of prediabetes among the TyG index quartiles. TyG index: the triglyceride-glucose index. **Figure S2.** The cumulative hazard of prediabetes among the TyG-BMI quartiles. TyG-BMI: triglyceride glucose-body mass index. **Figure S3.** The cumulative hazard of prediabetes among the TG/HDL-C ratio quartiles. TG/HDL-C ratio: triglyceride/high-density lipoprotein cholesterol ratio. **Figure S4.** The cumulative hazard of prediabetes among the MetS-IR quartiles. MetS-IR: metabolic score for insulin resistance.

**Additional file 3:** STROBE_checklist_(S1 text).

## Data Availability

The data used in this study have been uploaded to the "Dryad" database (Chinese cohort: https://doi.org/10.5061/dryad.ft8750v; Japanese cohort: https://doi.org/10.5061/dryad.8q0p192).

## Abbreviations

IR: Insulin resistance
TyG index: The triglyceride-glucose index
TyG-BMI: Triglyceride glucose-body mass index
TG/HDL-C ratio: Triglyceride/high-density lipoprotein cholesterol ratio
MetS-IR: Metabolic score for insulin resistance
HIEC: Hyperinsulinemic-euglycemic clamp
TG: Triglyceride
FPG: Fasting plasma glucose
BMI: Body mass index
HR: Hazard ratio
CI: Confidence interval
AUC: Area under the curve
ROC: Receiver operating characteristic curve
S/DBP: Systolic/diastolic blood pressure
TC: Total cholesterol
TG: Triglyceride
LDL-C: Low-density lipid cholesterol
ALT: Alanine aminotransferase
AST: Aspartate aminotransferase
SD: Standard deviation
