# Supplementary material for: Predictive value of insulin resistance surrogates for the development of diabetes in individuals with baseline normoglycemia: findings from two independent cohort studies in China and Japan
Source: Diabetol Metab Syndr. 2024 Mar 16;16:68. doi: 10.1186/s13098-024-01307-x (PMC10943817; doi:10.1186/s13098-024-01307-x)
Supplement: Supplementary file 1 — Additional file 1: Table S1. Collinearity diagnostics steps of TyG index with other covariates. Table S2. Collinearity diagnostics steps of TyG-BMI with other covariates. Table S3. Collinearity diagnostics steps of TG/HDL-C ratio with other covariates. Table S4. Collinearity diagnostics steps of MetS-IR with other covariates. Table S5. Univariate Cox regression analysis between all baseline indicators and diabetes risk. Table S6. Best threshold and areas under the time-dependent receiver operating characteristic curves for TyG index, TyG-BMI, TyG-WC, MetS-IR, TG/HDL-C ratio predicting future diabetes risk in the Japanese cohort. [file 13098_2024_1307_MOESM1_ESM.docx]

Additional file Table S1: Collinearity diagnostics steps of TyG index with other covariates.

|  | Variance inflation factor | | | |
| --- | --- | --- | --- | --- |
|  | Step 1 | Step 2 | Step 3 | Step 4 |
| TyG index | 6.2 | 6.2 | 6.2 | 6.2 |
| Age | 1.4 | 1.4 | 1.4 | 1.4 |
| Sex | 2.4 | 2.4 | 2.4 | 2.4 |
| Race | 2 | 2 | 2 | 1.8 |
| Height | 49.4 | 2.2 | 2.2 | 2.2 |
| Weight | 160.7 | NA | NA | NA |
| BMI | 93.8 | 1.6 | 1.6 | 1.6 |
| SBP | 2.5 | 2.5 | 2.5 | 2.5 |
| DBP | 2.3 | 2.3 | 2.3 | 2.3 |
| FPG | 1.4 | 1.4 | 1.4 | 1.4 |
| TC | 9.1 | 9.1 | NA | NA |
| TG | 4.9 | 4.9 | 4.5 | 4.5 |
| HDL-C | 2 | 2 | 1.3 | 1.3 |
| LDL-C | 7.7 | 7.7 | 1.2 | 1.2 |
| ALT | 3.4 | 3.3 | 3.3 | 3.3 |
| AST | 3.1 | 3.1 | 3.1 | 3.1 |
| Drinking status | 7.5 | 7.5 | 7.5 | NA |
| Smoking status | 6.9 | 6.9 | 6.8 | 1.5 |

Abbreviations as in Table ​1.

Note-1: Variance inflation factor = 1/(1-R^2^).

Note-2: The variables with Variance inflation factor >5 will be regarded as collinear variables and cannot be included in the multiple regression model.

Additional file Table S2: Collinearity diagnostics steps of TyG-BMI with other covariates.

|  | Variance inflation factor | | | | |
| --- | --- | --- | --- | --- | --- |
|  | Step 1 | Step 2 | Step 3 | Step 4 | Step 5 |
| TyG-BMI | 43.2 | 43.1 | 2.5 | 2.5 | 2.5 |
| Age | 1.4 | 1.4 | 1.4 | 1.3 | 1.3 |
| Sex | 2.4 | 2.4 | 2.4 | 2.4 | 2.4 |
| Race | 1.9 | 1.9 | 1.9 | 1.9 | 1.7 |
| Height | 49.4 | 2.2 | 2.2 | 2.2 | 2.2 |
| Weight | 160.6 | NA | NA | NA | NA |
| BMI | 116.4 | 26.9 | NA | NA | NA |
| SBP | 2.5 | 2.5 | 2.5 | 2.5 | 2.5 |
| DBP | 2.3 | 2.3 | 2.3 | 2.3 | 2.3 |
| FPG | 1.4 | 1.4 | 1.2 | 1.2 | 1.2 |
| TC | 9.1 | 9.1 | 9.1 | NA | NA |
| TG | 5.7 | 5.7 | 2.2 | 1.7 | 1.7 |
| HDL-C | 1.9 | 1.9 | 1.9 | 1.3 | 1.2 |
| LDL-C | 7.7 | 7.7 | 7.7 | 1.2 | 1.2 |
| ALT | 3.4 | 3.4 | 3.3 | 3.3 | 3.3 |
| AST | 3.1 | 3.1 | 3.1 | 3.1 | 3.1 |
| Drinking status | 7.5 | 7.5 | 7.5 | 7.5 | NA |
| Smoking status | 6.9 | 6.9 | 6.9 | 6.8 | 1.5 |

Abbreviations as in Table ​1.

Note-1: Variance inflation factor = 1/(1-R^2^).

Note-2: The variables with Variance inflation factor >5 will be regarded as collinear variables and cannot be included in the multiple regression model.

Additional file Table S3: Collinearity diagnostics steps of TG/HDL-C ratio with other covariates.

|  | Variance inflation factor | | | | |
| --- | --- | --- | --- | --- | --- |
|  | Step 1 | Step 2 | Step 3 | Step 4 | Step 5 |
| TG/HDL-C ratio | 14.4 | 14.4 | 2 | 1.5 | 1.5 |
| Age | 1.4 | 1.4 | 1.4 | 1.3 | 1.3 |
| Sex | 2.4 | 2.4 | 2.4 | 2.4 | 2.3 |
| Race | 1.9 | 1.9 | 1.8 | 1.8 | 1.7 |
| Height | 49.5 | 2.2 | 2.2 | 2.2 | 2.2 |
| Weight | 161.2 | NA | NA | NA | NA |
| BMI | 94 | 1.5 | 1.5 | 1.5 | 1.5 |
| SBP | 2.5 | 2.5 | 2.5 | 2.5 | 2.5 |
| DBP | 2.3 | 2.3 | 2.3 | 2.3 | 2.3 |
| FPG | 1.2 | 1.2 | 1.2 | 1.2 | 1.2 |
| TC | 9.3 | 9.3 | 9.3 | NA | NA |
| TG | 12.6 | 12.5 | NA | NA | NA |
| HDL-C | 2.9 | 2.9 | 2.4 | 1.4 | 1.4 |
| LDL-C | 7.9 | 7.9 | 7.9 | 1.2 | 1.2 |
| ALT | 3.4 | 3.3 | 3.3 | 3.3 | 3.3 |
| AST | 3.1 | 3.1 | 3.1 | 3.1 | 3.1 |
| Drinking status | 7.6 | 7.6 | 7.5 | 7.5 | NA |
| Smoking status | 6.9 | 6.9 | 6.9 | 6.8 | 1.5 |

Abbreviations as in Table ​1.

Note-1: Variance inflation factor = 1/(1-R^2^).

Note-2: The variables with Variance inflation factor >5 will be regarded as collinear variables and cannot be included in the multiple regression model.

Additional file Table S4: Collinearity diagnostics steps of MetS-IR with other covariates.

|  | Variance inflation factor | | | | |
| --- | --- | --- | --- | --- | --- |
|  | Step 1 | Step 2 | Step 3 | Step 4 | Step 5 |
| MetS-IR | 58 | 56.9 | 2.8 | 2.7 | 2.7 |
| Age | 1.4 | 1.4 | 1.4 | 1.3 | 1.3 |
| Sex | 2.4 | 2.4 | 2.4 | 2.4 | 2.4 |
| Race | 1.9 | 1.9 | 1.8 | 1.8 | 1.7 |
| Height | 50.2 | 2.2 | 2.2 | 2.2 | 2.2 |
| Weight | 163.7 | NA | NA | NA | NA |
| BMI | 111.3 | 31.9 | NA | NA | NA |
| SBP | 2.5 | 2.5 | 2.5 | 2.5 | 2.5 |
| DBP | 2.3 | 2.3 | 2.3 | 2.3 | 2.3 |
| FPG | 1.4 | 1.4 | 1.2 | 1.2 | 1.2 |
| TC | 9.1 | 9.1 | 9.1 | NA | NA |
| TG | 4.3 | 4.3 | 2 | 1.6 | 1.6 |
| HDL-C | 6.9 | 6.8 | 2.4 | 1.6 | 1.6 |
| LDL-C | 7.7 | 7.7 | 7.6 | 1.2 | 1.2 |
| ALT | 3.4 | 3.4 | 3.3 | 3.3 | 3.3 |
| AST | 3.1 | 3.1 | 3.1 | 3.1 | 3.1 |
| Drinking status | 7.6 | 7.6 | 7.5 | 7.5 | NA |
| Smoking status | 6.9 | 6.9 | 6.9 | 6.8 | 1.5 |

Abbreviations as in Table ​1.

Note-1: Variance inflation factor = 1/(1-R^2^).

Note-2: The variables with Variance inflation factor >5 will be regarded as collinear variables and cannot be included in the multiple regression model.

Additional file Table S5: Univariate Cox regression analysis between all baseline indicators and diabetes risk.

|  | HR (95% CI) (Per SD increase) | *P* |
| --- | --- | --- |
| Age | 2.34 (2.26, 2.41) | <0.0001 |
| Sex |  |  |
| Male | 1.0 |  |
| Female | 0.49 (0.45, 0.53) | <0.0001 |
| Height (cm) | 1.07 (1.03, 1.10) | 0.0004 |
| Weight (kg) | 1.83 (1.78, 1.89) | <0.0001 |
| BMI (kg/m^2^) | 2.00 (1.95, 2.05) | <0.0001 |
| SBP (mmHg) | 1.89 (1.83, 1.94) | <0.0001 |
| DBP (mmHg) | 1.66 (1.61, 1.71) | <0.0001 |
| FPG (mmol/L) | 4.40 (4.26, 4.55) | <0.0001 |
| TC (mmol/L) | 1.25 (1.21, 1.29) | <0.0001 |
| TG (mmol/L) | 1.29 (1.28, 1.30) | <0.0001 |
| HDL-C (mml/L) | 0.76 (0.73, 0.79) | <0.0001 |
| LDL-C (mmol/L) | 1.15 (1.11, 1.19) | <0.0001 |
| ALT (IU/L) | 1.09 (1.09, 1.10) | <0.0001 |
| AST (IU/L) | 1.08 (1.07, 1.09) | <0.0001 |
| Drinking status |  |  |
| No | 1.0 |  |
| Yes | 1.04 (0.90, 1.19) | 0.6133 |
| Not record | 2.14 (1.96, 2.34) | <0.0001 |
| Smoking status |  |  |
| No | 1.0 |  |
| Yes | 1.77 (1.57, 2.00) | <0.0001 |
| Not record | 2.67 (2.42, 2.96) | <0.0001 |

HR: Hazard ratio; CI: confidence interval; SD: standard deviation; other abbreviations as in Table ​1.

Additional file Table S6: Best threshold and areas under the time-dependent receiver operating characteristic curves for TyG index, TyG-BMI, TyG-WC, MetS-IR, TG/HDL-C ratio predicting future diabetes risk in the Japanese cohort.

|  | AUC (best threshold) | | | |
| --- | --- | --- | --- | --- |
| Predict time | TyG index | TyG-BMI | TG/HDL-C ratio | MetS-IR |
| 1-year | 0.73276(8.06527) | 0.76808(168.92292) | 0.70982(0.65042) | 0.78184(37.1745) |
| 2-years | 0.66527(8.4621) | 0.65732(159.67648) | 0.60608(1.01221) | 0.68824(35.9742) |
| 3-years | 0.70882(8.42858) | 0.7075(210.12806) | 0.6731(0.65672) | 0.70978(35.9873) |
| 4-years | 0.70161(8.22604) | 0.68051(203.24187) | 0.6693(0.62129) | 0.69387(35.9873) |
| 5-years | 0.70419(8.23204) | 0.70602(201.40869) | 0.67677(0.62129) | 0.71189(33.9097) |
| 6-years | 0.7129(8.23204) | 0.72176(201.40869) | 0.69201(0.59825) | 0.73083(33.7715) |
| 7-years | 0.7265(8.22818) | 0.73917(200.23018) | 0.70271(0.59086) | 0.74731(33.9275) |
| 8-years | 0.72682(8.22791) | 0.73953(200.23018) | 0.69925(0.59086) | 0.74583(31.9678) |
| 9-years | 0.72098(8.20111) | 0.72978(196.49102) | 0.68738(0.61177) | 0.73524(31.7261) |
| 10-years | 0.71218(8.21243) | 0.73035(196.49102) | 0.68046(0.60918) | 0.73693(31.7902) |
| 11-years | 0.71199(8.20111) | 0.7314(182.88649) | 0.67103(0.69476) | 0.73267(31.7902) |
| 12-years | 0.6996(8.21243) | 0.71476(182.60332) | 0.65279(0.72764) | 0.71886(31.7902) |
| 13-years | 0.62717(8.47637) | 0.6012(184.22098) | 0.61616(0.88681) | 0.62252(30.9591) |

Abbreviations: AUC: area under the curve; other abbreviations as in table 1.
