# Supplementary material for: Predictive value of insulin resistance surrogates for the development of diabetes in individuals with baseline normoglycemia: findings from two independent cohort studies in China and Japan
Source: Diabetol Metab Syndr. 2024 Mar 16;16:68. doi: 10.1186/s13098-024-01307-x (PMC10943817; doi:10.1186/s13098-024-01307-x)
Supplement: Supplementary file 2 — Additional file 2: Figure S1. The cumulative hazard of prediabetes among the TyG index quartiles. TyG index: the triglyceride-glucose index. Figure S2. The cumulative hazard of prediabetes among the TyG-BMI quartiles. TyG-BMI: triglyceride glucose-body mass index. Figure S3. The cumulative hazard of prediabetes among the TG/HDL-C ratio quartiles. TG/HDL-C ratio: triglyceride/high-density lipoprotein cholesterol ratio. Figure S4. The cumulative hazard of prediabetes among the MetS-IR quartiles. MetS-IR: metabolic score for insulin resistance. [file 13098_2024_1307_MOESM2_ESM.docx]

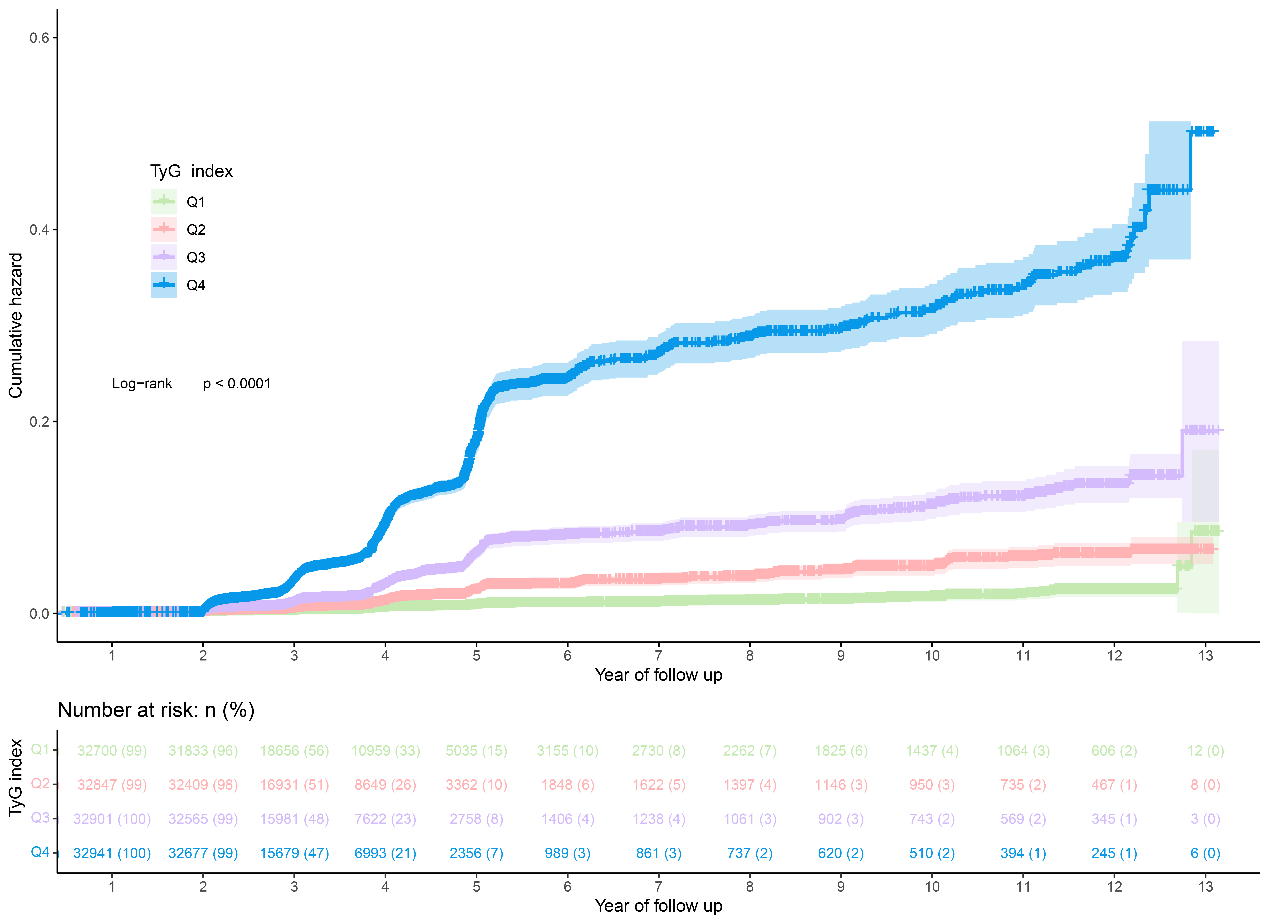


**Additional file Figure S1:** The cumulative hazard of prediabetes among the TyG index quartiles. TyG index: the triglyceride-glucose index.


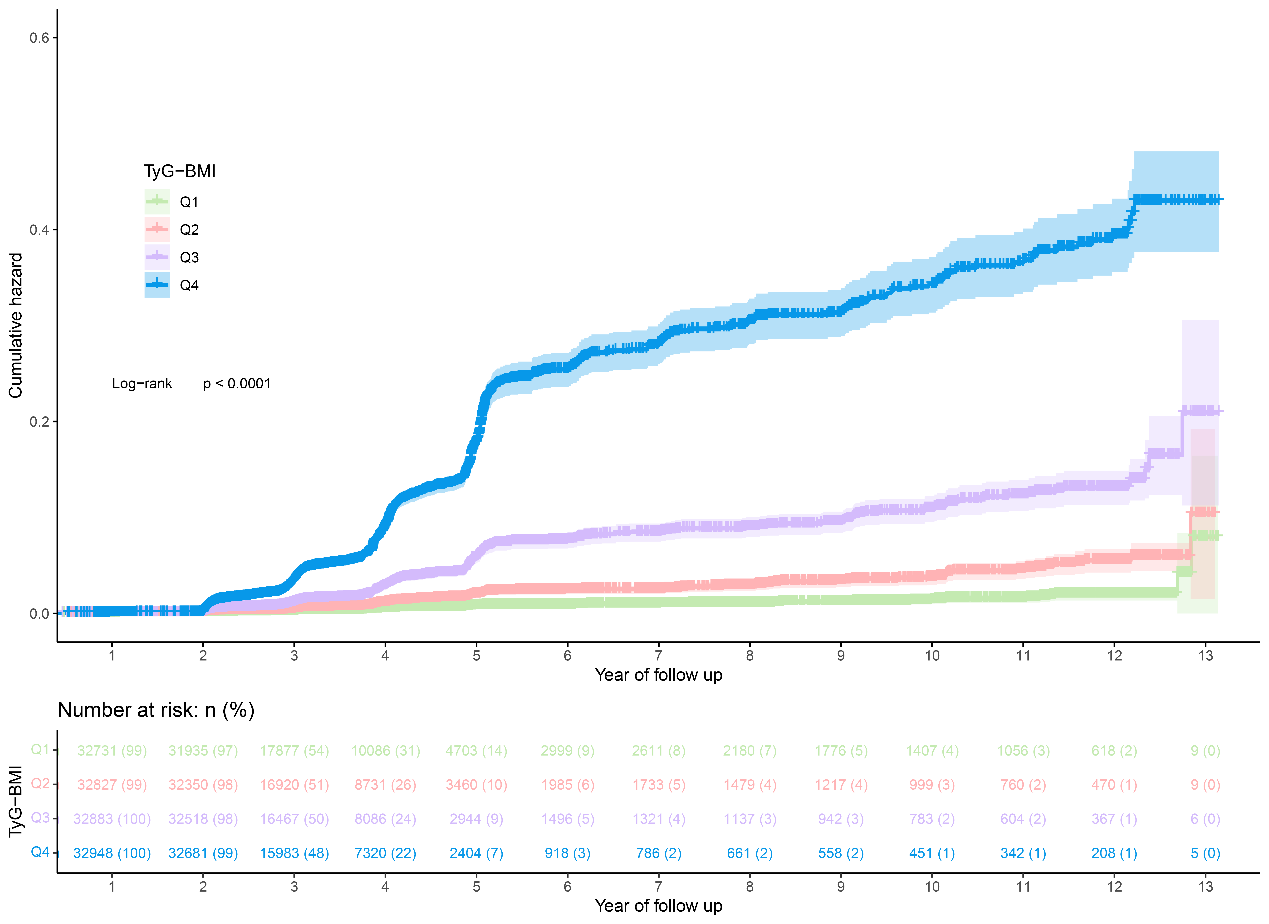


**Additional file Figure S2:** The cumulative hazard of prediabetes among the TyG-BMI quartiles. TyG-BMI: triglyceride glucose-body mass index.


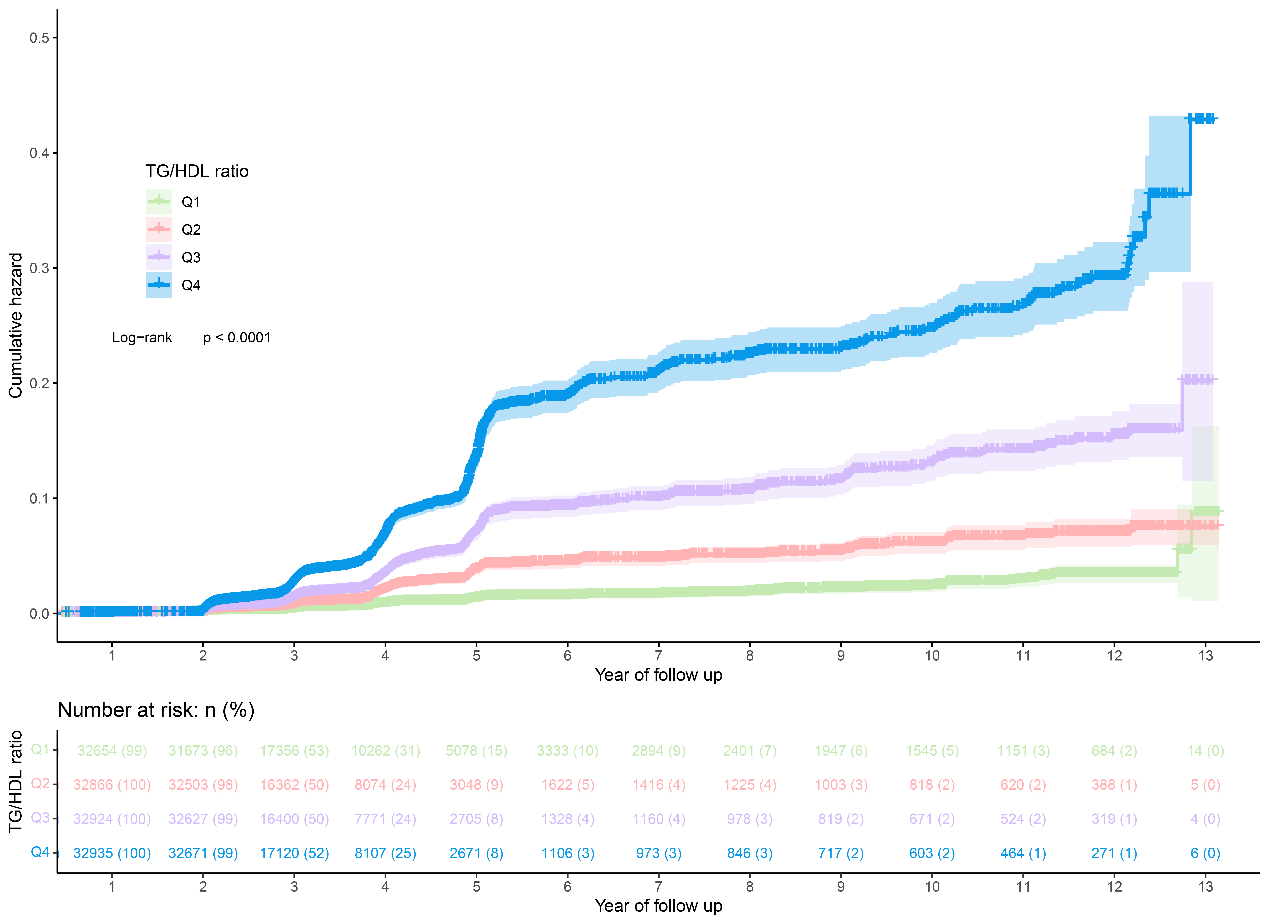


**Additional file Figure S3:** The cumulative hazard of prediabetes among the TG/HDL-C ratio quartiles. TG/HDL-C ratio: triglyceride/ high-density lipoprotein cholesterol ratio.


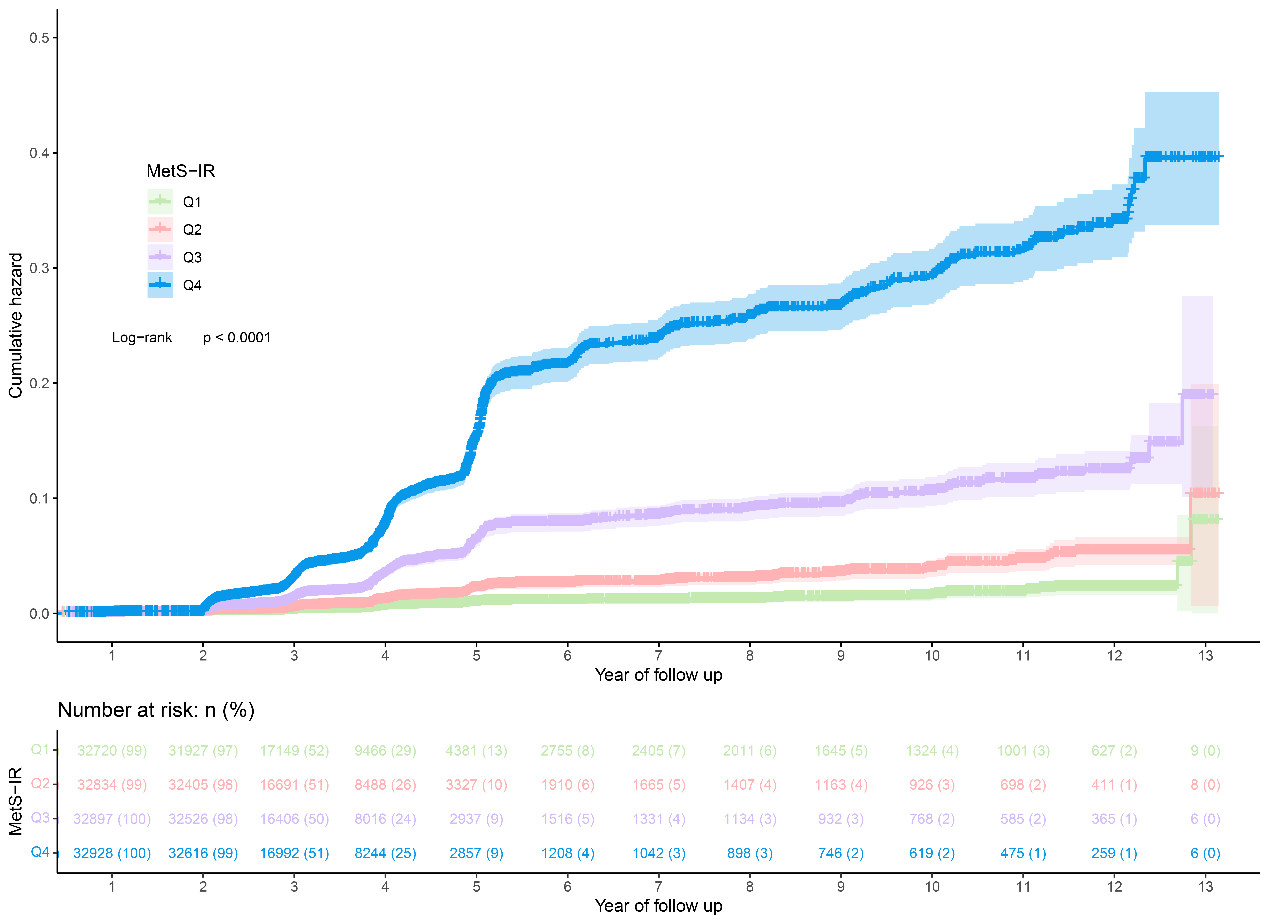


**Additional file Figure S4:** The cumulative hazard of prediabetes among the MetS-IR quartiles. MetS-IR: metabolic score for insulin resistance.
